# Sleep medicine in Saudi Arabia: evolution, current landscape, and future directions a state-of-the-art review with an embedded health-services analysis of Ministry of Health sleep centers, 2022 to 2025

**DOI:** 10.3389/frsle.2026.1941767

**Published:** 2026-09-15

**Authors:** Mana Alshahrani, Ashah Jman, Abdallah Alsoheimi, Salim Baharoon

**Affiliations:** 1 King Fahd Medical City, Riyadh, Saudi Arabia; 2 Alfaisal University College of Medicine, Riyadh, Saudi Arabia; 3 Ministry of Health, Riyadh, Saudi Arabia

**Keywords:** CPAP, health services research, health workforce, obstructive sleep apnea, polysomnography, road safety, Saudi Arabia, sleep medicine

## Abstract

**Background:**

Sleep medicine in Saudi Arabia has developed from a single academic laboratory in the late 1990s into an emerging national service. That development has played out against a heavy and still under-recognized burden of obstructive sleep apnea (OSA), low public awareness, and rising national attention to prevention, access, road safety, and value-based care.

**Objective:**

To describe the evolution and current landscape of sleep medicine in Saudi Arabia, document the Ministry of Health (MOH) National Sleep Medicine Program delivered during 2022 to 2025, and interpret remaining service gaps against Vision 2030 priorities and international benchmarks.

**Methods:**

We conducted a state-of-the-art narrative review and an ecological health-services analysis of routinely collected MOH cluster sleep-center returns. The operational dataset included 26 cluster-year observations across eight clusters and 41 standardized indicators. We summarized trends using counts, proportions, population-adjusted rates, and the compound annual growth rate (CAGR) defined as (ending value / beginning value)^∧^(1/years) minus 1. Spearman rank correlations explored associations between infrastructure, workforce, and output, with ties handled by mid-rank assignment. A capacity-to-demand gap model estimated annual diagnostic need under base-case and sensitivity scenarios. Program milestones were triangulated against MOH documents, Saudi Commission for Health Specialties (SCFHS) records, Saudi epidemiologic studies, AASM standards, and WHO and OECD reports.

**Results:**

Active MOH sleep centers grew from five to eight between 2022 and 2025. PSG bed capacity rose from 8 to 20 (CAGR 35.7%), and annual sleep-study output increased from 2,290 to 5,851 (CAGR 36.7%). Pediatric in-lab PSG accounted for 557 studies in 2025, or 17% of in-lab activity. Workforce growth lagged infrastructure (CAGR 14.9%); MOH sleep-physician density reached 0.42 per million served population, far below reported international ratios. The Program delivered a national master plan, a Virtual Health Hospital sleep clinic, a readiness manual, MOH classification of eight centers, public- and private-sector regulatory frameworks, a traffic-safety proposal, public-awareness activity, and a national training workshop. Under the base case (12% adult OSA prevalence, 65% adult-population fraction, 5% annual diagnostic capture), the MOH network met about 4% of estimated annual need; sensitivity scenarios produced a coverage range of roughly 3 to 11%. The CPAP backlog-to-fulfillment ratio was 7:1, and PSG wait times varied 68-fold across centers.

**Conclusions:**

Saudi sleep medicine has moved quickly from an academic subspecialty to a nationally organized service. The next phase should shift from infrastructure expansion alone to a learning health-system model built around a national sleep registry, workforce planning, centralized PAP procurement and adherence support, standardized triage, and integration with chronic-disease, occupational-health, and road-safety programs.

## 1 Introduction

Sleep disorders shape cardiovascular, metabolic, neurocognitive, and occupational outcomes, and they intersect with road safety in ways that few other chronic conditions do (Sateia, 2014). The third edition of the International Classification of Sleep Disorders catalogs more than 80 distinct entities, yet obstructive sleep apnea (OSA) continues to drive the majority of service demand in most health systems (Sateia, 2014). In Saudi Arabia, several factors plausibly amplify that burden: a high prevalence of obesity and metabolic disease, an aging population, rapid urbanization, and a labor market that depends on commercial driving and aviation (Wali et al., 2017; BaHammam et al., 2008). Saudi population-based work places adult OSA prevalence at 8.8% to 12.8%, with figures above 30% among middle-aged men and among adults with obesity, type 2 diabetes, or resistant hypertension (Wali et al., 2017; BaHammam et al., 2009). Awareness has not kept pace. In the largest Saudi survey to date, only 12.9% of 16,662 adults demonstrated good knowledge of OSA (Aldhahir et al., 2025).

The clinical and public-health consequences of untreated OSA are substantial. Untreated OSA carries an approximately two- to threefold higher risk of incident stroke, heart failure, atrial fibrillation, resistant hypertension, and motor-vehicle crashes (Yaggi et al., 2005; Tregear et al., 2009). Saudi and regional studies have tied driver sleepiness to roughly 11 to 12% of road traffic accidents, with similar concerns documented among Gulf commercial pilots and workers on extended or rotating shifts (Almeneessier and BaHammam, 2019; Aljurf and Olaish, 2018; Alhejaili et al., 2021; Barger et al., 2005). A meaningful share of that risk is modifiable. Observational and meta-analytic evidence link adherent continuous positive airway pressure (CPAP) therapy to lower cardiovascular events, lower all-cause mortality, and fewer sleepiness-related collisions, although the absolute size of the effect varies by study design and case mix (Patil et al., 2019; Marin et al., 2005).

Against that background, Saudi sleep medicine has progressed from a single academic sleep laboratory in the late 1990s to a structured service that now operates across MOH clusters, university hospitals, the National Guard Health Affairs, the Ministry of Defense, and the private sector (BaHammam, 2011; Almeneessier, 2017). The trajectory is timely. Vision 2030 and the Health Sector Transformation Program have placed access, prevention, digital health, and value-based care at the center of health-system reform (Saudi Vision, 2021; Ministry of Health, Kingdom of Saudi Arabia, 2024), Sleep medicine maps naturally onto those priorities, because the discipline links diagnostic capacity to chronic-disease prevention, workplace safety, and measurable outcomes. This review reconstructs the development of the specialty, analyzes four years of MOH operational data, summarizes the National Sleep Medicine Program, and proposes priorities for the next phase of national scale-up.

## 2 Methods

We designed the article as a state-of-the-art review with an embedded ecological health-services analysis. The narrative component synthesized Saudi and international literature on sleep-medicine epidemiology, workforce, service models, accreditation, and policy. The health-services component drew on routinely collected, aggregated MOH cluster sleep-center returns covering 2022 to 2025. No patient-level identifiable information was accessed or analyzed.

The operational dataset comprised 26 cluster-year observations from eight MOH clusters and 41 standardized indicators covering center status, PSG beds, home sleep apnea testing (HSAT) devices, adult and pediatric sleep studies, wait times, workforce, PAP prescriptions, and PAP waiting lists. Data cleaning and harmonization were carried out before analysis to align indicator definitions across reporting years.

For each capacity, workforce, and output indicator, we calculated descriptive statistics (counts, proportions, and population-adjusted rates) and the CAGR over the 4 year window. CAGR was defined as (ending value / beginning value)^∧^(1/n) - 1, where n is the number of years between the first and last reporting periods. Bivariate associations between infrastructure (PSG beds, HSAT devices), workforce (sleep physicians and technologists), and output (in-lab PSG, home studies, total studies) were explored with Spearman rank correlations; tied ranks were handled by mid-rank assignment, and significance was assessed at a two-sided alpha of 0.05.

We modeled the gap between current capacity and estimated annual diagnostic need using a base case and a sensitivity analysis. The base case assumed an adult OSA prevalence of 12% (the midpoint of published Saudi estimates), an adult-population fraction of 65% of the resident population, and an annual diagnostic-capture target of 5% of the prevalent pool. Two sensitivity scenarios bracketed plausible alternatives: a conservative scenario (8.8% prevalence, 60% adult fraction, 3% capture) and an expansive scenario (19.5% prevalence, 70% adult fraction, 7% capture). Population denominators were taken from General Authority for Statistics (GASTAT) mid-year estimates for 2022 to 2025 [General Authority for Statistics (GASTAT), Kingdom of Saudi Arabia, 2025] Coverage is reported as the ratio of observed MOH study output to estimated need for each scenario.

Program-level descriptions were triangulated against MOH planning documents, SCFHS training and workforce records, Saudi epidemiologic studies, AASM accreditation standards, and WHO and OECD policy reports. Because the analysis used aggregated administrative data, the findings should be read as a service-level assessment, not an individual-level outcomes study, and the capacity-to-demand model should be interpreted as a planning estimate that depends on the underlying assumptions made transparent above. Calculations were performed in standard spreadsheet and statistical software.

### 2.1 Literature search strategy

We searched PubMed/MEDLINE, Scopus, Web of Science, and Embase from January 2000 to June 2026, supplemented by hand-searching of the reference lists of key articles and of official reports from the Saudi MOH, the Saudi Commission for Health Specialties, the Council of Health Insurance, the American Academy of Sleep Medicine, the World Health Organization, and the OECD. Search terms combined controlled vocabulary and free text: (“sleep medicine” OR “obstructive sleep apnea” OR “polysomnography” OR “insomnia” OR “narcolepsy”) AND (“Saudi Arabia” OR “Gulf” OR “health services” OR “workforce” OR “accreditation” OR “telemedicine”). Inclusion criteria were peer-reviewed studies, official health-authority reports, and clinical practice guidelines relevant to sleep-medicine epidemiology, service delivery, workforce, or policy. Exclusion criteria were non-English and non-Arabic records without translation, conference abstracts without full text, and opinion pieces without primary data. Two reviewers screened titles, abstracts, and full texts, resolving disagreements by consensus. Because this is a state-of-the-art narrative review, methodological quality was appraised with the Scale for the Assessment of Narrative Review Articles (SANRA) rather than a systematic-review risk-of-bias tool; the STROBE checklist governs the embedded ecological health-services analysis.

### 2.2 Statistical analysis

For the core indicators we report means with standard deviations (SD) and medians with interquartile ranges (IQR). Bivariate associations were quantified with the Spearman rank-correlation coefficient (rho) as the effect-size metric, with bootstrap 95% confidence intervals (CIs; 2,000 resamples) and exact two-sided p-values; ties were handled by mid-rank assignment. Between-year comparisons of waiting times used the Mann-Whitney U test. Because the ecological sample comprises eight clusters, confidence intervals are wide, and all inferential estimates are interpreted as hypothesis-generating rather than confirmatory. A full statistical appendix (means, SDs, medians, IQRs, correlation coefficients with 95% CIs, and exact p-values) is provided as Supplementary Table S1.

## 3 Historical background

Saudi sleep medicine originated at King Khalid University Hospital (KKUH), King Saud University, in Riyadh, where the first dedicated sleep laboratory opened in the late 1990s (BaHammam, 2011; Almeneessier, 2017). Early activity centered on attended PSG for tertiary referrals with suspected OSA. In the decade that followed, the field grew through academic consolidation rather than formal system design. No Saudi sleep-medicine fellowship existed, and early clinicians entered the field either through overseas fellowships or by combining sleep practice with their training in pulmonary medicine, internal medicine, family medicine, pediatrics, neurology, or psychiatry (Almeneessier, 2017).

The Saudi Sleep Medicine Society (SSMS) was formally registered in the third quarter of 2024 as the standalone professional body for the discipline, building on the Saudi Sleep Disorders Group, which had operated under the Saudi Thoracic Society since the early 2000s (Almeneessier, 2017). The SSMS now supports scientific meetings, Arabic-language patient education, and locally adapted clinical protocols [Almeneessier, 2017; Saudi Sleep Medicine Society (SSMS)., 2024]. In parallel, Saudi investigators built the local evidence base, beginning with descriptive work on snoring and sleep disorders (Wali et al., 1999) and progressing to population-based studies of OSA risk in Saudi men and women (BaHammam et al., 2008, 2009).

Regulatory recognition followed in stages. In 2009, the SCFHS accredited the first structured sleep medicine fellowship at King Saud University, which remained the only training site for several years before expansion to the King Faisal Specialist Hospital and Research Center, King Fahad Medical City, and King Abdulaziz University Hospital (Saudi Commission for Health Specialties, 2023). By the late 2010s, sleep medicine had moved from a small academic practice into a recognized and regulated service line.

## 4 Growth phase, 2009 to 2018

The period between the 2009 SCFHS fellowship accreditation and the opening of the first MOH cluster sleep centers marks the geographic expansion of the specialty beyond Riyadh. Epidemiologic studies clarified the burden of OSA, while bariatric, cardiometabolic, and ENT services generated rising referral volume (Wali et al., 2017; BaHammam et al., 2008, 2009). New MOH cluster centers opened in the Eastern Province, with the Dammam center opening in 2012; at King Fahad Medical City within the Riyadh Second cluster in 2014; and at King Abdullah Medical City within the Makkah cluster in 2018, creating the first geographically distributed public-sector footprint for sleep medicine in the Kingdom.

Training capacity matured in parallel. By the early 2020s, six SCFHS-accredited fellowship programs were producing about 15 Saudi sleep physicians every 2 years, a substantial increase over the earlier single-center model (Saudi Commission for Health Specialties, 2023; Ministry of Health, Kingdom of Saudi Arabia, 2026). Saudi-trained sleep technologists also began entering clinical practice, reducing but not eliminating reliance on expatriate recruitment. Awareness campaigns coordinated by the MOH, the SSMS, and academic centers raised the visibility of snoring, daytime sleepiness, and OSA in public discourse [Saudi Sleep Medicine Society (SSMS)., 2024].

That growth phase coincided with the launch of Saudi Vision 2030 in 2016 and with the Health Sector Transformation Program (Saudi Vision, 2021; Ministry of Health, Kingdom of Saudi Arabia, 2024). Sleep medicine is not named explicitly in the Vision 2030 documents, but its priorities (access, prevention of non-communicable disease, digital health, and outcome-based commissioning) are directly relevant to the clinical model. The cluster-based reorganization of MOH care produced, for the first time, an administrative structure within which sleep services could be planned, monitored, and benchmarked (Al-Hanawi et al., 2019).

## 5 The current landscape, 2022 to 2025

### 5.1 Sleep centers and services

Operational returns from MOH cluster sleep centers show a clear expansion of national capacity (Figure 1, Table 1). Active MOH sleep centers grew from five to eight between 2022 and 2025, with new services in Riyadh First (pediatrics), Hail, and Jazan joining established centers in Eastern (Dammam), Riyadh Second, Qassim, Al-Ahsa, and Makkah (Ministry of Health, Kingdom of Saudi Arabia, 2026). PSG bed capacity rose from 8 to 20 beds (CAGR 35.7%), whereas HSAT device capacity grew more modestly from 12 to 16 (CAGR 10.1%). Annual sleep-study output approached 5,900 studies (CAGR 36.7%). Population-adjusted output rose from 7.1 to 16.6 studies per 100,000, a 134% absolute increase that remains well below international benchmarks (American Academy of Sleep Medicine, 2023; Watson et al., 2017).

**Figure 1:**
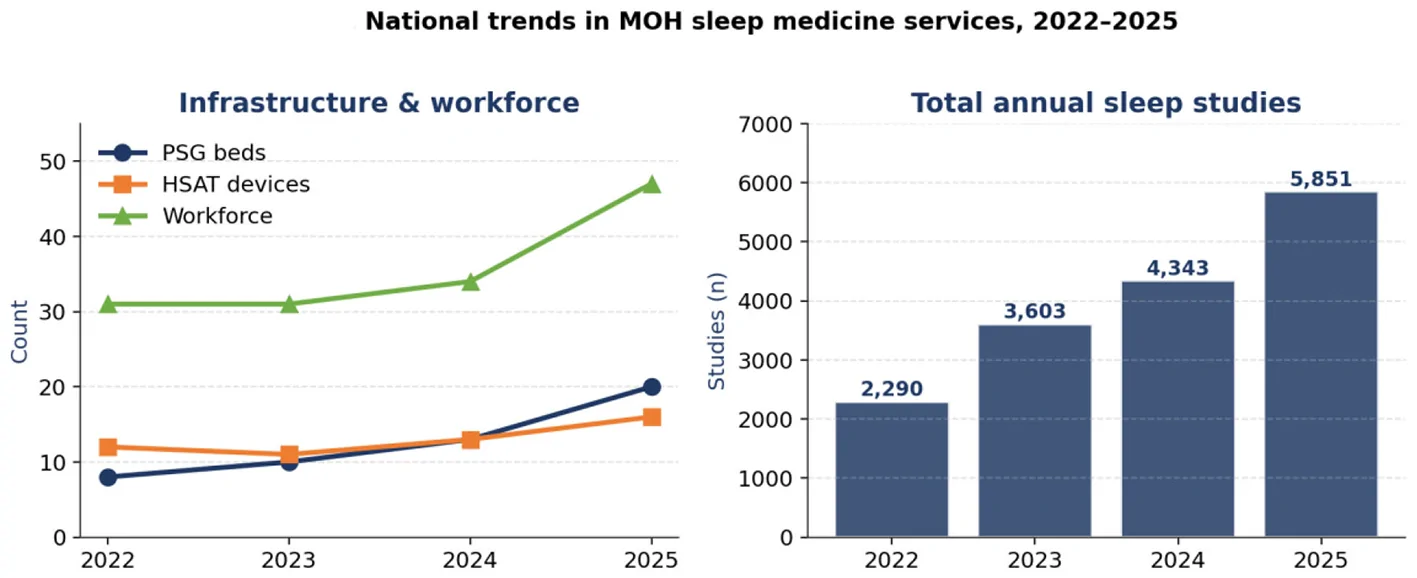
National trends in MOH sleep medicine services, 2022 to 2025. Source: MOH National Sleep Medicine Program operational returns (Ministry of Health, Kingdom of Saudi Arabia, 2026).

**Table 1:** National MOH sleep medicine capacity and output, 2022 to 2025.

Indicator	2022	2023	2024	2025	CAGR 2022 to 2025
Active centers (*n*)	5	5	6	8	n/a
PSG beds (*n*)	8	10	13	20	+35.7%
HSAT devices (*n*)	12	11	13	16	+10.1%
Adult sleep physicians	10	10	12	12	+6.3%
Pediatric sleep physicians	2	2	2	3	+14.5%
Sleep technologists	19	19	20	32	+19.0%
Total workforce	31	31	34	47	+14.9%
In-lab studies (PSG)	1,296	3,153	2,470	3,294	+36.5%
Home studies (HSAT + APAP)	1,707	829	3,199	3,453	+26.5%
Total sleep studies	2,290	3,603	4,343	5,851	+36.7%
Studies per 100,000 population	7.1	11.0	12.4	16.6	n/a
PSG beds per million	0.25	0.31	0.37	0.57	n/a
Sleep physicians per million	0.37	0.37	0.40	0.42	n/a

MOH cluster operational returns, 2022 to 2025; GASTAT mid-year population estimates [Ministry of Health, Kingdom of Saudi Arabia, 2026; General Authority for Statistics (GASTAT), Kingdom of Saudi Arabia, 2025]. CAGR, compound annual growth rate; PSG, polysomnography; HSAT, home sleep apnea test; APAP, auto-titrating positive airway pressure.

Interpretation (Figure 1): The near-tripling of study output against a 134% rise in population-adjusted capacity indicates genuine efficiency gains rather than passive growth. Clinically, however, the absolute rate of 16.6 studies per 100,000 remains an order of magnitude below the throughput of mature programs, so growth from a low base should not be mistaken for adequacy. Across the 26 cluster-year observations, the mean annual sleep-study volume per cluster was 731 (SD 812; median 304, IQR 188 to 899), reflecting marked right-skew driven by the Riyadh Second reference center.

Activity remained highly concentrated (Table 2). In 2025, Riyadh Second performed 3,411 studies (about 58% of all MOH activity), followed by the Eastern Province (Figure 2) (Ministry of Health, Kingdom of Saudi Arabia, 2026). Pediatric sleep medicine emerged as a defined service line in 2025, driven by the new Riyadh First center, which contributed 315 of the 557 national pediatric in-lab PSGs. That brought pediatric activity to 17% of in-lab studies, within the 10% to 20% range reported internationally (Marcus et al., 2012). When non-MOH facilities are included, Saudi Arabia currently hosts about 23 active sleep centers and 58 sleep medicine consultants (Almeneessier, 2017).

**Table 2:** Cluster-level service capacity and 2025 output.

Cluster	Tier	Start year	PSG beds	HSAT devices	Workforce	Studies 2025	PSG wait (days)
Riyadh Second (KFMC)	A	2014	6	5	21	3,411	450
Eastern (Dammam)	A	2012	4	4	4	899	479
Qassim	B	2022	1	2	4	386	30
Riyadh First (KSMC)	A	2025	4	1	6	317	21
Al-Ahsa	B	2022	2	0	4	304	198
Hail	B	2024	1	1	2	272	7
Makkah	B	2018	1	2	3	188	300
Jazan	B	2025	1	1	3	74	270

MOH cluster operational returns, 2025 (Ministry of Health, Kingdom of Saudi Arabia, 2026). Tier A, high-volume reference centers; Tier B, regional or newly inaugurated centers. KFMC, King Fahad Medical City; KSMC, King Saud Medical City.

**Figure 2:**
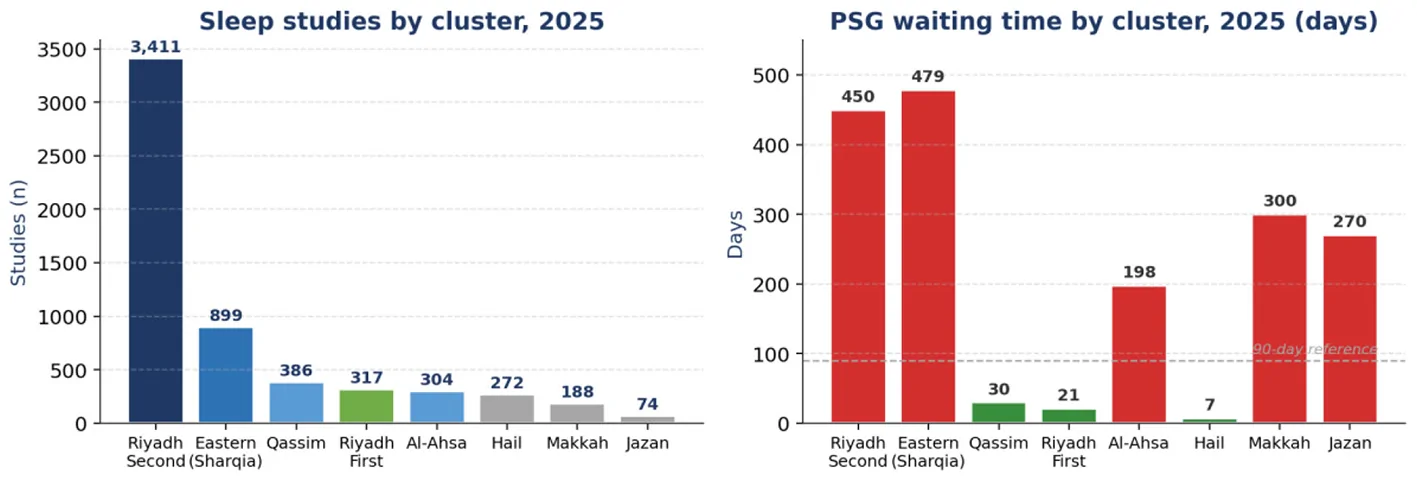
Cluster-level service volume **(left)** and PSG wait-time inequity **(right)** in 2025. The 90-day reference line approximates international service standards (England, 2022).

Infrastructure and output were strongly associated: PSG bed count correlated with in-lab study output (Spearman rho = 0.86; bootstrap 95% CI 0.71 to 0.94; *p* < 0.001), HSAT device count with home-study output (rho = 0.83; 95% CI 0.66 to 0.92; *p* < 0.001), and total workforce with total study volume (rho = 0.62; 95% CI 0.30 to 0.82; *p* = 0.001). These associations indicate that installed capacity is being used rather than left idle, and that workforce is the more elastic constraint.

Interpretation (Figure 2): The juxtaposition of a single dominant center with a 68-fold variation in waiting time is the clearest signal of inequity in the system. National PSG waiting time did not improve significantly between 2022 and 2025 (median 220 vs 114 days; Mann-Whitney U, *p* = 0.83). Clinically, patients with severe OSA in high-wait clusters such as Makkah and the Eastern Province, where waits exceed one year, accrue avoidable cardiovascular and road-safety risk during the delay, which is the strongest argument for risk-based rather than geographically fixed triage.

### 5.2 Workforce and training

In 2025 the MOH network employed 15 sleep physicians (12 adult and three pediatric), 32 sleep technologists, and a small number of allied staff for an attributed population of about 35.3 million [Ministry of Health, Kingdom of Saudi Arabia, 2026; General Authority for Statistics (GASTAT), Kingdom of Saudi Arabia, 2025]. That corresponds to 0.42 sleep physicians per million MOH-served population. The estimated all-sector Saudi ratio is approximately 0.10 per 100,000, markedly below reported ratios in the United States, Germany, Canada, and Japan (Figure 3) (American Academy of Sleep Medicine, 2023; Watson et al., 2017; Penzel et al., 2021). We note that the all-sector figure is an internal program estimate derived from MOH and SSMS workforce records rather than a published census, and should be interpreted as such. Saudization of the technologist workforce remained near 44% across the 4 year window, indicating that recent growth has depended substantially on expatriate recruitment (Ministry of Health, Kingdom of Saudi Arabia, 2026).

**Figure 3:**
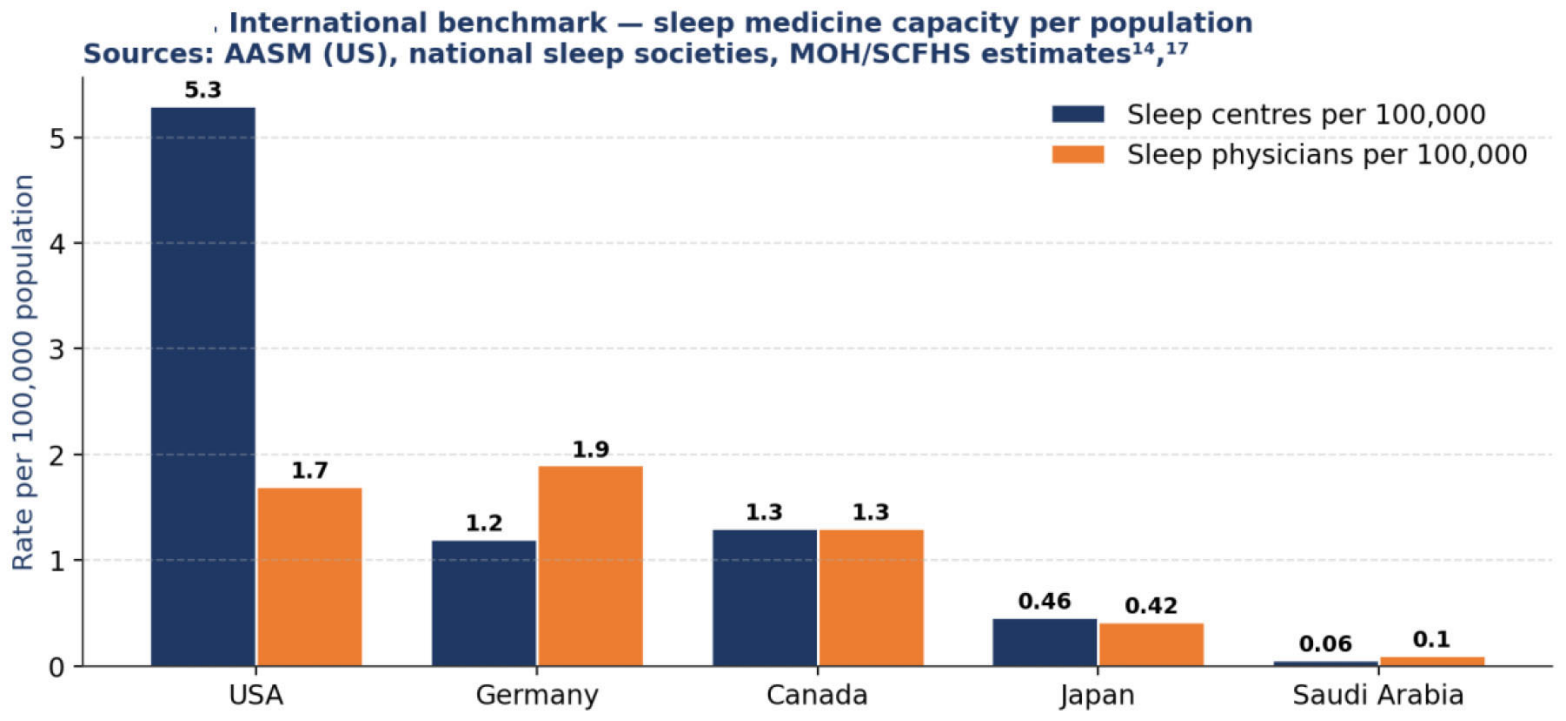
International benchmark comparing Saudi sleep medicine capacity (centers per 100,000 and sleep physicians per 100,000) against the United States, Germany, Canada, and Japan (American Academy of Sleep Medicine, 2023; Watson et al., 2017; Penzel et al., 2021).

The composition of the workforce is heterogeneous. Of approximately 58 Saudi sleep specialists, the largest specialty backgrounds are internal medicine (*n* = 25), pediatrics (*n* = 8), family medicine (*n* = 8), ENT (*n* = 5), psychiatry (*n* = 2), and neurology (*n* = 1). Nine certified sleep technicians support clinical operations (Almeneessier, 2017). Forty-five practitioners hold a medical fellowship in sleep medicine, with smaller numbers holding higher diplomas, training-program certificates, or technologist diplomas (Almeneessier, 2017). Behavioral sleep medicine providers, particularly psychologists delivering cognitive behavioral therapy for insomnia (CBT-I), are notably absent from formal workforce reporting; that is a structural gap given the central role of those clinicians in international service models (Sawyer et al., 2011).

Interpretation The workforce is dominated by fellowship-trained physicians from internal-medicine backgrounds, with a near-absence of behavioral sleep-medicine providers. This composition directly explains the OSA-centric service model and the under-development of insomnia and behavioral service lines. The stagnant Saudization rate (about 44%) signals that the expansion has been achieved through expatriate recruitment rather than a self-sustaining national pipeline.

### 5.3 Regulation and accreditation

The SCFHS provides the principal regulatory framework. It governs recognition of sleep medicine as a subspecialty, accredits training programs, and certifies sleep technologists (Saudi Commission for Health Specialties, 2023). The Council of Health Insurance regulates reimbursement codes for diagnostic studies and CPAP therapy (Council of Health, Insurance, and Kingdom of Saudi Arabia, 2024). A defining development of the current phase has been the operationalization of the first MOH sleep-center classification framework (see Section 6.3). Between January and December 2025, eight MOH sleep centers received formal MOH classification certificates, two at Tier A (Riyadh Second / KFMC and Qassim) and six at Tier B (Makkah, Al-Ahsa, Dammam, Hail, Riyadh First, and Jazan) (Ministry of Health, Kingdom of Saudi Arabia, 2026). That is the first Saudi-specific accreditation framework dedicated to sleep medicine, and it provides a foundation for alignment with AASM standards (American Academy of Sleep Medicine, 2023).

### 5.4 Clinical service models

The MOH network operates three diagnostic pathways: attended in-lab PSG, HSAT, and auto-titrating positive airway pressure (APAP) trial. The lab-vs.-home split has been variable across the reporting years, reflecting operational fluctuations at the dominant center rather than coordinated national policy. In 2025 the distribution stabilized at 41% in-lab and 59% home, which is consistent with mature international service models in which HSAT serves as the primary screening pathway and in-lab PSG is reserved for higher-complexity cases (Marcus et al., 2012; Sawyer et al., 2011).

CPAP remains the cornerstone of OSA therapy. In 2025, 326 CPAP devices were prescribed across the reporting MOH centers against a documented waiting list of 2,393 patients, a backlog-to-fulfillment ratio of roughly 7:1 (Figure 4) (Ministry of Health, Kingdom of Saudi Arabia, 2026). The BiPAP backlog stood at 151 patients against 43 prescriptions. The recent decision by two large centers to move PAP device supply outside the sleep-medicine cost center highlights the absence of a single national procurement pathway, and represents the most pressing operational discontinuity in the current system.

**Figure 4:**
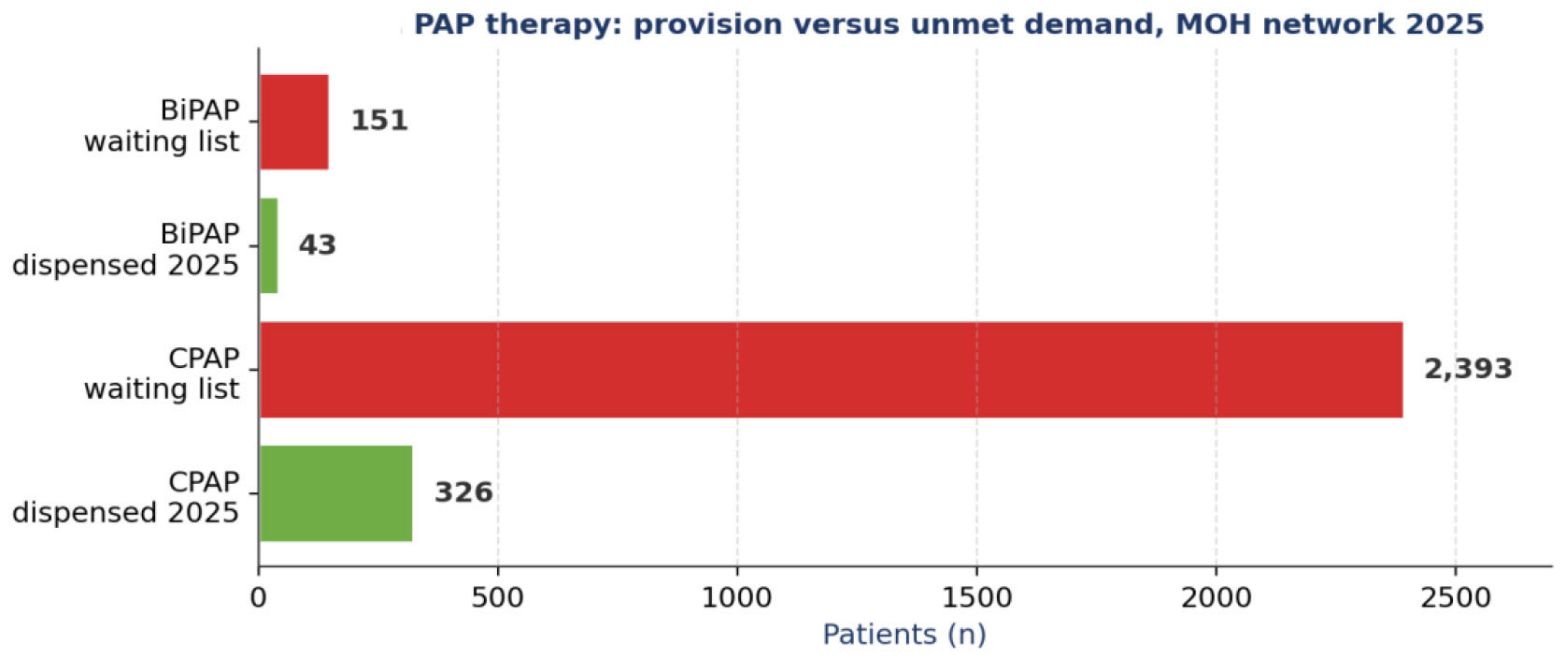
PAP therapy provision against unmet demand within the MOH sleep medicine network, 2025. The CPAP backlog-to-fulfillment ratio is approximately 7:1 (Ministry of Health, Kingdom of Saudi Arabia, 2026).

Interpretation (Figure 4): The 7:1 backlog visually conveys that diagnosis has become decoupled from treatment. Because untreated OSA carries measurable cardiovascular, metabolic, and motor-vehicle-crash risk, this backlog represents deferred clinical harm rather than a purely administrative queue. Mature programs report CPAP adherence at 30 and 90 days as a core outcome; the MOH network cannot yet do so, which precludes outcome-level benchmarking and reinforces the case for a centralized procurement and adherence program.

### 5.5 The broader spectrum of sleep disorders and their management

Although OSA dominates current MOH service activity, the clinical remit of sleep medicine spans the full ICSD-3 spectrum, and the OSA dominance seen here is a service-configuration finding rather than a true reflection of disease burden. Chronic insomnia is the most prevalent sleep disorder in the general population; cognitive behavioral therapy for insomnia (CBT-I) is the guideline-endorsed first-line treatment, yet it is not represented in current MOH operational reporting and no CBT-I-trained behavioral workforce is recorded. Regional post-pandemic data underline this burden: in one 403-participant survey from the wider region, 48.4% of respondents met criteria for insomnia, with OSA risk stratified by the STOP-BANG instrument (Hasan and Aladdin, 2024). Central sleep apnea, frequently secondary to heart failure or opioid use, requires distinct pathways, including optimization of the underlying condition and, in selected non-heart-failure phenotypes, adaptive servo-ventilation. Restless legs syndrome and periodic limb movement disorder require iron-status assessment and, where indicated, alpha-2-delta ligands or cautious dopaminergic therapy. The parasomnias, including REM sleep behavior disorder (a recognized prodrome of alpha-synucleinopathy), and the central disorders of hypersomnolence, including narcolepsy, require specialized diagnostic and therapeutic pathways; our dataset already captures a rising national narcolepsy caseload (141 cases under follow-up in 2025), managed with wake-promoting agents and, where available, oxybate therapy. The near-absence of insomnia, RLS, parasomnia, and circadian-rhythm service lines in MOH operational reporting is therefore a structural gap and a priority for national service diversification.

## 6 Vision 2030 and the MOH national sleep medicine program

Saudi Vision 2030 set explicit priorities for healthcare: improved access, a value-based and prevention-oriented model, and digitally enabled service delivery (Saudi Vision, 2021). The Health Sector Transformation Program operationalized those priorities by reorganizing MOH care into clusters, establishing the Health Holding Company, and redefining the MOH as a regulator and policymaker rather than a direct service operator (Ministry of Health, Kingdom of Saudi Arabia, 2024; Al-Hanawi et al., 2019). Within that framework the MOH General Directorate of Specialized Centers, together with the SSMS, has delivered a coordinated program since 2024 organized around eight pillars, collectively the National Sleep Medicine Master Plan (Ministry of Health, Kingdom of Saudi Arabia, 2026; Saudi Sleep Medicine Society (SSMS)., 2024). The pillars are summarized below.

### 6.1 Pillar 1: national sleep medicine master plan

The MOH formally launched the Sleep Medicine Centers Project in 2024. The Plan rests on four elements: a scientific advisory team of sleep medicine experts, four strategic objectives anchored to the Health Sector Transformation Program, a comprehensive baseline analysis of infrastructure and referral pathways across all healthcare clusters, and a national committee tasked with developing and validating clinical protocols (Ministry of Health, Kingdom of Saudi Arabia, 2026; Saudi Sleep Medicine Society (SSMS)., 2024). It represents the first national strategic framework dedicated to sleep medicine within an MOH transformation program in the Eastern Mediterranean Region.

### 6.2 Pillar 2: enhancing access to specialized sleep services

Three operational instruments drove access expansion. An MOH Specialized-Centers Readiness Manual for Sleep Medicine Centers was developed and distributed to all clusters; the manual sets out scope of service, organizational structure, staffing plans, governmental licensure pathways, readiness checklists, clinical practice guidelines, clinical pathways, credentialing standards, and key-performance-indicator manuals (Ministry of Health, Kingdom of Saudi Arabia, 2026). At the same time, the MOH issued formal instructions to establish sleep medicine centers in five priority regions (Jeddah, Al-Jouf, Jazan, Asir, and Tabuk), extending coverage into previously under-served geographies (Ministry of Health, Kingdom of Saudi Arabia, 2026). A specialized sleep medicine clinic was also opened within the Virtual Health Hospital to provide remote consultations to clusters with workforce shortfalls, leveraging the same digital-health infrastructure that scaled MOH telehealth from 1,326 virtual appointments in 2022 to 4,559 in 2024 (Ministry of Health, Kingdom of Saudi Arabia, 2026; Al-Hanawi et al., 2019).

### 6.3 Pillar 3: MOH-classified sleep medicine facilities

Between January and December 2025, the MOH General Directorate of Specialized Centers issued formal classification certificates to eight MOH sleep medicine centers after on-site survey and standards-compliance verification (Table 3). Two centers achieved Tier A: King Fahad Medical City (Riyadh Second cluster) and King Fahad Specialist Hospital (Qassim). Six centers achieved Tier B: King Abdullah Medical City (Makkah), King Fahad Hospital (Al-Ahsa), King Fahad Specialist Hospital (Dammam), King Salman Specialist Hospital (Hail), King Saud Medical City (Riyadh First, pediatric), and Jazan Specialist Hospital (Ministry of Health, Kingdom of Saudi Arabia, 2026). The cycle represents the first Saudi-issued accreditation dedicated to sleep medicine facilities.

**Table 3:** MOH-classified sleep medicine facilities, 2025 (Pillar 3 of the National Sleep Medicine Master Plan).

Facility (cluster)	City	Tier	Validity	Effective from
King Fahad Medical City (Riyadh Second)	Riyadh	A	1 year	27 Jan 2025
King Fahad Specialist Hospital (Qassim)	Buraidah	A	1 year	1 Apr 2025
King Saud Medical City, pediatric (Riyadh First)	Riyadh	A	1 year	11 Nov 2025
King Abdullah Medical City (Makkah)	Makkah	B	1 year	1 Mar 2025
King Fahad Specialist Hospital (Dammam)	Dammam	B	1 year	May 2025
King Fahad Hospital (Al-Ahsa)	Al-Ahsa	B	1 year	1 May 2025
King Salman Specialist Hospital (Hail)	Hail	B	1 year	1 Jun 2025
Jazan Specialist Hospital	Jazan	B	1 year	10 Dec 2025

MOH General Directorate of Specialized Centers classification certificates issued in 2025 (Ministry of Health, Kingdom of Saudi Arabia, 2026). Validity is for one calendar year, renewable subject to standards-compliance survey.

### 6.4 Pillar 4: improving quality and efficiency through regulation

Two regulatory instruments were developed in tandem. A framework for sleep medicine centers within MOH healthcare clusters now defines the standards, scope, and governance applicable to public facilities (Ministry of Health, Kingdom of Saudi Arabia, 2026). A complementary framework for the private sector was developed and submitted to the MOH General Directorate of Licensing, addressing a long-standing governance gap that had previously allowed unregulated private sleep services to operate with variable quality (Ministry of Health, Kingdom of Saudi Arabia, 2026). Together, the two instruments are intended to align diagnostic and therapeutic standards across the Saudi sleep medicine ecosystem.

### 6.5 Pillar 5: public awareness and prevention

Expert-led media engagement was an explicit component of the Master Plan. The National Lead for Sleep Medicine and the SSMS partnered with the MOH social-media channels @LiveWellMOH and @SaudiMOH to produce bilingual content on practical sleep advice, OSA-attributable cardiovascular risk, and the social and occupational consequences of unrecognized sleep disorders (Ministry of Health, Kingdom of Saudi Arabia, 2026). Awareness materials were produced and distributed to primary-care and chronic-disease clinics. The activity directly addresses the awareness gap documented by Aldhahir and colleagues, who reported good knowledge of OSA in only 12.9% of a 16,662-adult national sample (Aldhahir et al., 2025).

### 6.6 Pillar 6: official World Sleep Day recognition

In March 2024, Saudi Arabia, represented by the National Lead for Sleep Medicine, received official recognition from the World Sleep Society for its national World Sleep Day awareness campaign on the theme “Sleep equity for global health.” (Ministry of Health, Kingdom of Saudi Arabia, 2026; World Sleep Society, 2025). The recognition formalizes Saudi participation in international sleep-medicine advocacy (Asian Society of Sleep Medicine, 2025).

### 6.7 Pillar 7: sleep and traffic safety integration

Sleep-attributable road traffic accidents are an established but under-addressed problem in the Kingdom. Saudi data attribute roughly 11% to 12% of road traffic accidents to driver sleepiness, with up to 30% of near-miss events similarly attributable (Almeneessier and BaHammam, 2019). Untreated OSA increases motor-vehicle-crash risk approximately 2.5-fold, and treatment with PAP has been associated with substantial attenuation of that excess risk in meta-analytic data, although the magnitude varies by adherence and case mix (Tregear et al., 2009). In 2024, the MOH National Sleep Medicine Program prepared and submitted a formal Sleep Disorders and Traffic Safety project proposal to the Ministerial Committee for Traffic Safety. The proposal recommends systematic screening of commercial drivers and structured re-licensing pathways for drivers with diagnosed OSA on PAP therapy, (Ministry of Health, Kingdom of Saudi Arabia, 2026) and aligns with Vision 2030 road-safety targets and WHO Global Road Safety guidance (World Health Organization, 2023).

### 6.8 Pillar 8: training, education, and value-based care

A national Sleep Medicine Workshop was convened in Riyadh on 21 and 22 December 2024 for sleep physicians, sleep laboratory specialists, pulmonologists, primary-care and family physicians, pediatricians, psychiatrists, neurologists, ENT specialists, nurses, and medical students (Ministry of Health, Kingdom of Saudi Arabia, 2026). In parallel, the MOH and the SCFHS have collaborated to develop a national professional certification pathway for sleep laboratory technicians, addressing the workforce bottleneck identified in Figure 5 and Section 7 (Saudi Commission for Health Specialties, 2023; Ministry of Health, Kingdom of Saudi Arabia, 2026). A value-based sleep medicine initiative, modeled on a national OSA-management program and developed with the National Health Insurance Center and a local industry partner, is in advanced design and will feed into MOH cost-calculation and value-based commissioning workstreams (Ministry of Health, Kingdom of Saudi Arabia, 2026).

**Figure 5:**
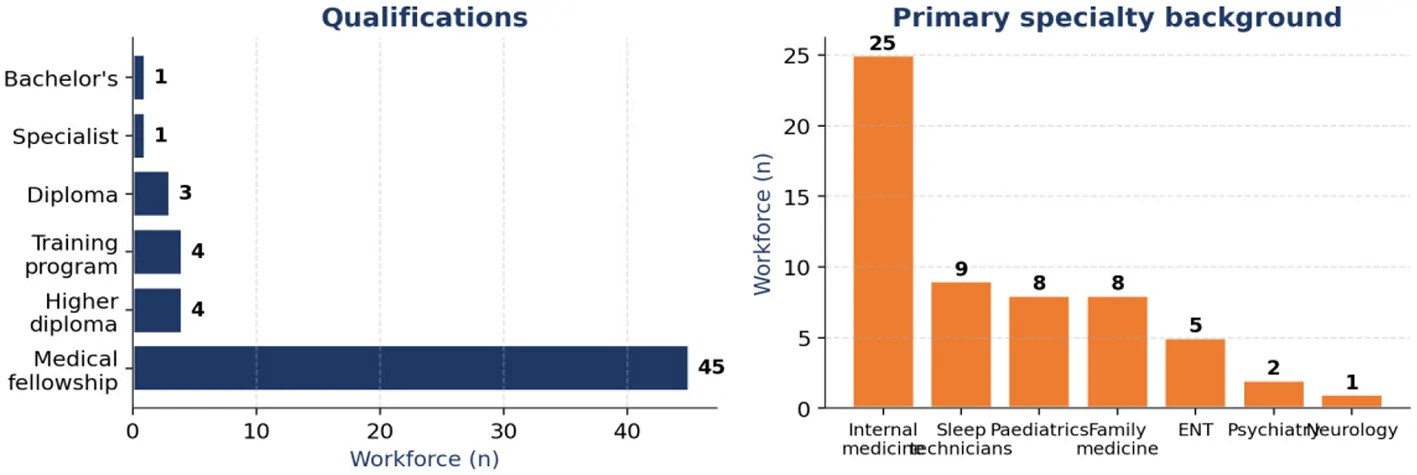
Saudi sleep medicine workforce by qualification and primary specialty background. Source: MOH and SSMS workforce census, 2024 to 2025 (Almeneessier, 2017; Ministry of Health, Kingdom of Saudi Arabia, 2026).

Interpretation (Figure 3) and standardized benchmarking: Expressed in the same standardized indicators used internationally, the MOH network delivered 16.6 studies per 100,000 population in 2025, against an estimated need of approximately 390 per 100,000 and against typical high-income-country throughput of several hundred per 100,000 per year. Saudi sleep-physician density (all-sector estimate approximately 0.10 per 100,000) is an order of magnitude below the United States (1.7), Germany (1.9), and Canada (1.3), and below Japan (0.42). National median PSG waiting time (114 days) sits at the upper limit of the NHS England 18-week (126-day) referral-to-treatment standard and far exceeds the AASM operational target of a clinic-to-test interval under 30 days for high-risk OSA. Whereas mature programs report structured 30-day and 90-day CPAP adherence, the MOH network does not yet capture adherence at all. These standardized comparisons position Saudi Arabia as an emerging but capacity-constrained program rather than a mature one, and identify workforce and adherence, not infrastructure, as the binding constraints.

## 7 Challenges and gaps

Six structural gaps continue to constrain progress.

### 7.1 Gap between capacity and disease burden

Under the base-case parameters described in Section 2 (12% adult OSA prevalence, 65% adult-population fraction, 5% annual diagnostic capture), the estimated MOH-attributable demand is approximately 137,670 studies per year. MOH study output in 2025 (5,851) represents 4.2% of that estimate (Ministry of Health, Kingdom of Saudi Arabia, 2026). Even with non-MOH capacity included, all-sector coverage is unlikely to exceed roughly 18% to 22% of estimated need, leaving a structural shortfall on the order of 100,000 studies per year. Sensitivity analysis bounds the picture without changing its shape. Under the conservative scenario (8.8% prevalence, 60% adult fraction, 3% capture), MOH coverage rises to about 11% of estimated need; under the expansive scenario (19.5% prevalence, 70% adult fraction, 7% capture), it falls to about 3%. Across all scenarios, the order of magnitude of the gap remains the same. Case-finding figures echo the diagnostic shortfall: roughly 40,000 of an estimated 3 million Saudi adults with OSA carry a formal diagnosis, and approximately 637 of an estimated 10,900 Saudi narcolepsy cases are formally identified. The narcolepsy denominator is extrapolated from an international point-prevalence estimate of about 0.04% and should be reconfirmed in Saudi data before it is used for service planning (Almeneessier, 2017).

### 7.2 Workforce capacity

Adult sleep physician headcount in the MOH network rose from 10 to 12 over 4 years (+20%), well below the growth rate of physical capacity (Ministry of Health, Kingdom of Saudi Arabia, 2026). Several clusters operate with a single sleep physician, which represents a continuity risk for the service. The fellowship pipeline now produces about 15 graduates every 2 years, (Saudi Commission for Health Specialties, 2023; Ministry of Health, Kingdom of Saudi Arabia, 2026) which remains below the estimated need of 50 to 70 net new sleep physicians by 2030 to reach a density of two per million.

Underlying causes: the shortage reflects several interacting mechanisms rather than a single deficit. Fellowship output is constrained by the small number of accredited training centers and supervising faculty; the technologist workforce depends heavily on expatriate recruitment (Saudization near 44%), which is vulnerable to localization policy and turnover; there is no protected sleep-medicine career track or dedicated staffing establishment within the cluster workforce model, so posts compete with other specialties; and behavioral sleep-medicine providers are not part of the recognized cadre at all. Together these explain why workforce, rather than physical infrastructure, is the rate-limiting step.

### 7.3 Access inequity

In 2025, PSG wait times in the MOH network ranged from 7 days (Hail) to 479 days (Eastern), a 68-fold spread that is geographically rather than clinically mediated (Figure 2) (Ministry of Health, Kingdom of Saudi Arabia, 2026). No national triage tool is currently in place to expedite high-risk patients such as commercial drivers, patients with severe nocturnal hypoxemia, or pediatric OSA with growth failure. Services remain absent in five major regions despite documented case loads. The diagnostic delay for narcolepsy in Saudi Arabia is not yet established in published Saudi data; the often-cited 8- to 10-year interval is derived from international cohorts (Almeneessier, 2017) and should be reconfirmed locally before it is used as a planning benchmark (Ministry of Health, Kingdom of Saudi Arabia, 2026).

Underlying causes: the disparities are largely explained by the historical concentration of tertiary investment and specialist workforce in Riyadh and the Eastern Province, combined with geographically (rather than clinically) determined referral, so that reference centers accumulate demand while newer peripheral centers remain under-utilized. In the absence of risk-based triage and inter-cluster load-balancing, a patient's waiting time is determined by their catchment rather than their clinical acuity, which is the mechanism underlying the 68-fold range.

### 7.4 PAP supply and consumables

The CPAP backlog of 2,393 patients is the single most urgent operational gap because it weakens the link between diagnosis and clinical benefit (Figure 4) (Ministry of Health, Kingdom of Saudi Arabia, 2026). Untreated OSA is associated with cardiovascular, metabolic, neurocognitive, and road-safety harms, (Yaggi et al., 2005; Tregear et al., 2009) so a diagnosis that is not followed by timely PAP initiation represents deferred clinical value for the patient and the health system.

Underlying causes: the backlog is driven by fragmented procurement. PAP supply is not commissioned as a single national treatment program; two large centers have moved device supply outside the sleep-medicine cost center; and reimbursement pathways for consumables are inconsistent between clusters. As a result, diagnostic throughput structurally outpaces treatment delivery at a ratio of roughly 7 to 1, and the constraint is a supply-chain and commissioning problem rather than a clinical one.

### 7.5 Narrow clinical model and absence of a national registry

The MOH network is functionally an OSA service. CBT-I, recommended as first-line therapy for chronic insomnia by both the AASM and the American College of Physicians, (Qaseem et al., 2016; Edinger et al., 2021) is not represented in operational reporting. Restless legs syndrome, parasomnias, circadian rhythm disorders, obesity hypoventilation syndrome, and surgical and dental sleep medicine are similarly underdeveloped (Ministry of Health, Kingdom of Saudi Arabia, 2026). A more fundamental constraint is the absence of a national sleep registry. Without ICSD-3-coded diagnoses, apnea-hypopnea index severity, comorbidity, and PAP adherence data, the network cannot perform case-mix-adjusted benchmarking, demonstrate outcomes against international peers, or contribute robust national data to international research.

### 7.6 Public awareness

Public awareness remains a system-level constraint. With good OSA knowledge documented in only 12.9% of Saudi adults, (Aldhahir et al., 2025) many patients may not recognize symptoms or seek care until complications emerge. Expanding infrastructure without parallel health-literacy investment risks underuse of new centers in some regions and persistent diagnostic delay in others.

## 8 Future directions and strategic recommendations

Three principles should guide the next phase. Growth must be tied to measurable disease burden rather than to infrastructure targets alone. Workforce, PAP access, and adherence support should be treated as core capacity rather than as downstream operational details. Sleep medicine must be integrated into chronic-disease, occupational-health, and road-safety programs rather than left as a diagnostic silo. The recommendations that follow translate those principles into an implementable national agenda.

### 8.1 Emerging technologies: artificial intelligence, wearables, digital biomarkers, and telemedicine

Emerging technologies are reshaping sleep-medicine service delivery and are directly relevant to the Saudi capacity gap. Artificial-intelligence and machine-learning models can automate polysomnography scoring, triage high-risk patients, and predict disorders such as insomnia across demographic groups, potentially relieving the physician-scoring bottleneck identified in this review; however, models require local validation because performance can vary across demographic and device settings (Rashid et al., 2025). Consumer wearables and validated home sleep-apnea test devices extend screening beyond the laboratory and, coupled with central scoring, could unlock low-cost capacity in primary care. Digital biomarkers (from photoplethysmography, actigraphy, and mandibular-movement sensing) and cloud-based PAP telemonitoring enable remote adherence tracking, which is precisely the outcome dimension the MOH network currently cannot measure. Tele-sleep, already operational through the Virtual Health Hospital, provides the delivery backbone for these tools in under-served regions. Realizing this potential will require device validation in Saudi populations, clear regulatory pathways through the Saudi Food and Drug Authority, data-governance safeguards, and attention to digital equity so that technology narrows rather than widens regional disparities.

### 8.2 Recommendation 1: a national sleep medicine registry

Establish a mandatory minimum-dataset registry across MOH and academic sleep centers. Core fields should include ICSD-3-coded diagnosis, apnea-hypopnea index severity, body mass index, key comorbidities, study quality indicators, PAP initiation, 30 day and 90 day adherence, and patient-reported outcomes. A registry would enable case-mix-adjusted benchmarking, support value-based commissioning, and provide a platform for Saudi research output.

### 8.3 Recommendation 2: a unified national referral and triage pathway

Implement a Saudi-validated triage instrument at primary-care and specialty-entry points. The pathway should route uncomplicated high-probability OSA to HSAT, direct complex cardiopulmonary, neuromuscular, pediatric, or hypoventilation cases to in-lab PSG, and expedite high-risk groups such as commercial drivers, patients with severe nocturnal hypoxemia, and children with growth or neurocognitive concerns.

### 8.4 Recommendation 3: a national workforce plan

Set an explicit national target of at least two sleep physicians per million population by 2030. Achieving that target will require expanding SCFHS-accredited fellowship capacity, protecting sleep-medicine posts within the cluster workforce model, building defined career pathways for technologists, and embedding behavioral sleep medicine and CBT-I training into the service model.

### 8.5 Recommendation 4: centralized PAP procurement and adherence program

Create a single national PAP pathway that covers procurement, fitting, education, 30-day and 90-day adherence verification, telemonitoring, troubleshooting, and structured re-issue. The pathway should be commissioned as a treatment program rather than as isolated device purchasing, because the clinical value of PAP depends on timely initiation and sustained adherence.

### 8.6 Recommendation 5: HSAT scale-up via primary care

Scale HSAT through primary-care clusters using central scoring, specialist sign-off, and clear exclusion criteria. The approach would expand diagnostic throughput at relatively low cost while preserving in-lab PSG for complex cases, pediatrics, parasomnias, hypoventilation, severe comorbidity, and inconclusive home studies.

### 8.7 Recommendation 6: tele-sleep extension to under-served regions

Use the Virtual Health Hospital sleep clinic as the national backbone for tele-sleep in under-served regions, including Jeddah, Al-Jouf, Jazan, Asir, and Tabuk. Priority use cases include pre-test triage, post-test counseling, PAP adherence follow-up, and patient education, with escalation to in-person care when clinically indicated.

### 8.8 Recommendation 7: sleep and driving policy implementation

Operationalize the MOH Sleep Disorders and Traffic Safety proposal with the Ministerial Committee for Traffic Safety. Implementation should include risk-based screening for commercial drivers, standardized re-licensing criteria for treated OSA, documentation of PAP adherence, and a governance model that protects both patient confidentiality and public safety.

### 8.9 Recommendation 8: mature the Saudi accreditation framework

Develop the 2025 MOH classification framework into a mature Saudi accreditation pathway aligned with AASM principles. Annual reporting should include wait times, diagnostic yield, PAP initiation, adherence, complication rates, workforce coverage, and patient experience, so that accreditation becomes a driver of continuous improvement rather than a one-time certification exercise.

### 8.10 Recommendation 9: integration with chronic-disease programs

Embed sleep medicine in chronic-disease programs through bidirectional referral pathways with cardiology, endocrinology, obesity services, stroke clinics, bariatric surgery, mental health, and occupational medicine. That integration would shift sleep medicine from a referral-based specialty toward a preventive service line with measurable impact on cardiometabolic outcomes and on safety.

### 8.11 Recommendation 10: value-based commissioning and sustained public awareness

Complete and pilot the MOH and Council of Health Insurance value-based sleep medicine initiative, (Ministry of Health, Kingdom of Saudi Arabia, 2026, 2024) while sustaining bilingual awareness campaigns through MOH, SSMS, primary-care, and workplace-health channels. Outcomes should include diagnostic access, PAP initiation, adherence, patient-reported sleepiness, cardiovascular risk markers, and safety indicators.

### 8.12 Priorities for future research

Future research should move beyond descriptive service analysis in five directions. First, multicenter prospective longitudinal cohorts should link diagnosis to treatment initiation, adherence, and hard outcomes (cardiovascular events, motor-vehicle crashes, and glycemic control). Second, a national sleep registry with a standardized minimum dataset should be established as the data backbone for all subsequent work. Third, predictive artificial-intelligence models should be developed and locally validated for risk stratification, demand forecasting, no-show prediction, and automated PSG scoring (Rashid et al., 2025). Fourth, advanced health-services analytics, including data envelopment analysis and stochastic frontier analysis for center efficiency, time-series forecasting for capacity planning, and geospatial modeling of access equity, should be applied to guide investment. Fifth, pragmatic randomized and quasi-experimental studies of tele-sleep and centralized CPAP-adherence programs should test which interventions most efficiently convert diagnostic capacity into treated, adherent patients. Together, these would transform the national program from a service-reporting system into a learning health system.

## 9 Strengths and limitations

This review has three principal strengths. It combines historical synthesis with 4 years of MOH operational data, links service development to national transformation priorities, and translates observed gaps into policy-relevant recommendations. The analysis is grounded in routine service indicators, which makes the findings directly relevant to implementation rather than to abstract performance benchmarking.

Several limitations warrant emphasis. First, the design is ecological, at the cluster-year level, so associations cannot be interpreted causally and are subject to the ecological fallacy. Second, the analysis relies on aggregated administrative returns that were not collected for research; indicator definitions varied across years and centers, introducing potential reporting and misclassification bias (for example, the Makkah 2025 waiting time was recorded as “10” and reflected 10 months rather than 10 days, a discrepancy we identified and corrected against the source). Third, the dataset captures only the MOH sector, so national, all-sector inference is limited and non-MOH capacity was estimated rather than measured; the all-sector physician density (approximately 0.10 per 100,000) should be read as a planning estimate rather than a published rate. Fourth, the capacity-to-demand model depends on assumed parameters (12% adult OSA prevalence, 65% adult-population fraction, 5% annual diagnostic capture); we addressed this with a sensitivity analysis spanning conservative and expansive scenarios (Section 7.1), but the estimate remains a planning approximation. Fifth, case-mix variables (apnea-hypopnea index severity, comorbidity, body mass index), treatment adherence, and patient-reported outcomes were not available, precluding outcome-level analysis. Sixth, with only eight clusters, inferential statistics have limited power and wide confidence intervals, and international benchmarks may not fully account for differences in health-system structure, reimbursement, and population need. Finally, data derive from a single custodian, so independent verification was not possible. These limitations reinforce, rather than undermine, the central recommendation for a standardized national sleep registry.

## 10 Conclusion

Saudi sleep medicine has progressed in roughly 25 years from a single academic sleep laboratory at KKUH into a national service operating across eight MOH clusters and a broader multi-sector ecosystem. The 2022 to 2025 period was particularly important. PSG capacity and study output expanded, pediatric services emerged as a defined line, MOH center classification began, tele-sleep was introduced, and sleep medicine entered national conversations about prevention, occupational health, and road safety.

The same data show why the next phase must be more deliberate. The MOH network currently meets only a small fraction of estimated diagnostic need, workforce growth has not kept pace with infrastructure, PSG wait times vary widely by geography, and the PAP backlog limits the clinical value of diagnostic expansion. For Saudi sleep medicine to mature into a high-performing national program, the priority should now be to build a learning system: a registry, standardized triage, workforce expansion, centralized PAP access, accreditation-linked quality metrics, and integration with chronic-disease and safety agendas. That shift would position sleep medicine as a practical contributor to Vision 2030 goals for access, prevention, value, and population health.
