# Supplementary material for: Sleep medicine in Saudi Arabia: evolution, current landscape, and future directions a state-of-the-art review with an embedded health-services analysis of Ministry of Health sleep centers, 2022 to 2025
Source: Front Sleep. 2026 Sep 15;5:1941767. doi: 10.3389/frsle.2026.1941767 (PMC13621571; doi:10.3389/frsle.2026.1941767)
Supplement: Supplementary file 1 [file Table_1.docx]

**TABLES**

*Sleep Medicine in Saudi Arabia , Alshahrani et al., 2026*

This document contains the three tables referenced in the manuscript. Tables are provided in editable Word format per Karger requirements. Each table is on its own page.

**Table 1. National MOH sleep medicine capacity and output, 2022 to 2025.**

| **Indicator** | **2022** | **2023** | **2024** | **2025** | **CAGR 2022 to 2025** |
| --- | --- | --- | --- | --- | --- |
| Active centers (n) | 5 | 5 | 6 | 8 | n/a |
| PSG beds (n) | 8 | 10 | 13 | 20 | +35.7% |
| HSAT devices (n) | 12 | 11 | 13 | 16 | +10.1% |
| Adult sleep physicians | 10 | 10 | 12 | 12 | +6.3% |
| Pediatric sleep physicians | 2 | 2 | 2 | 3 | +14.5% |
| Sleep technologists | 19 | 19 | 20 | 32 | +19.0% |
| Total workforce | 31 | 31 | 34 | 47 | +14.9% |
| In-lab studies (PSG) | 1,296 | 3,153 | 2,470 | 3,294 | +36.5% |
| Home studies (HSAT + APAP) | 1,707 | 829 | 3,199 | 3,453 | +26.5% |
| Total sleep studies | 2,290 | 3,603 | 4,343 | 5,851 | +36.7% |
| Studies per 100,000 population | 7.1 | 11.0 | 12.4 | 16.6 | n/a |
| PSG beds per million | 0.25 | 0.31 | 0.37 | 0.57 | n/a |
| Sleep physicians per million | 0.37 | 0.37 | 0.40 | 0.42 | n/a |

*Sources: MOH cluster operational returns, 2022 to 2025; GASTAT mid-year population estimates (Refs. 20, 26). CAGR, compound annual growth rate; PSG, polysomnography; HSAT, home sleep apnea test; APAP, auto-titrating positive airway pressure.*

**Table 2. Cluster-level service capacity and 2025 output.**

| **Cluster** | **Tier** | **Start year** | **PSG beds** | **HSAT devices** | **Workforce** | **Studies 2025** | **PSG wait (days)** |
| --- | --- | --- | --- | --- | --- | --- | --- |
| Riyadh Second (KFMC) | A | 2014 | 6 | 5 | 21 | 3,411 | 450 |
| Eastern (Dammam) | A | 2012 | 4 | 4 | 4 | 899 | 479 |
| Qassim | B | 2022 | 1 | 2 | 4 | 386 | 30 |
| Riyadh First (KSMC) | A | 2025 | 4 | 1 | 6 | 317 | 21 |
| Al-Ahsa | B | 2022 | 2 | 0 | 4 | 304 | 198 |
| Hail | B | 2024 | 1 | 1 | 2 | 272 | 7 |
| Makkah | B | 2018 | 1 | 2 | 3 | 188 | 300 |
| Jazan | B | 2025 | 1 | 1 | 3 | 74 | 270 |

*Source: MOH cluster operational returns, 2025 (Ref. 20). Tier A, high-volume reference centers; Tier B, regional or newly inaugurated centers. KFMC, King Fahad Medical City; KSMC, King Saud Medical City. The Makkah 2025 PSG wait time reported as 10 in the source spreadsheet reflects 10 months and has been converted to 300 days for consistency.*

**Table 3. MOH-classified sleep medicine facilities, 2025 (Pillar 3 of the National Sleep Medicine Master Plan).**

| **Facility (cluster)** | **City** | **Tier** | **Validity** | **Effective from** |
| --- | --- | --- | --- | --- |
| King Fahad Medical City (Riyadh Second) | Riyadh | A | 1 year | 27 Jan 2025 |
| King Fahad Specialist Hospital (Qassim) | Buraidah | A | 1 year | 1 Apr 2025 |
| King Saud Medical City, pediatric (Riyadh First) | Riyadh | A | 1 year | 11 Nov 2025 |
| King Abdullah Medical City (Makkah) | Makkah | B | 1 year | 1 Mar 2025 |
| King Fahad Specialist Hospital (Dammam) | Dammam | B | 1 year | May 2025 |
| King Fahad Hospital (Al-Ahsa) | Al-Ahsa | B | 1 year | 1 May 2025 |
| King Salman Specialist Hospital (Hail) | Hail | B | 1 year | 1 Jun 2025 |
| Jazan Specialist Hospital | Jazan | B | 1 year | 10 Dec 2025 |

*Source: MOH General Directorate of Specialized Centers classification certificates issued in 2025 (Ref. 20). Validity is for one calendar year, renewable subject to standards-compliance survey.*
